# The Burden of Image Based Emphysema and Bronchiolitis in HIV-Infected Individuals on Antiretroviral Therapy

**DOI:** 10.1371/journal.pone.0109027

**Published:** 2014-10-29

**Authors:** Giovanni Guaraldi, Giulia Besutti, Riccardo Scaglioni, Antonella Santoro, Stefano Zona, Ligabue Guido, Alessandro Marchioni, Gabriella Orlando, Federica Carli, Bianca Beghe, Leonardo Fabbri, Jonathon Leipsic, Don D. Sin, S. F. Paul Man

**Affiliations:** 1 Modena and Reggio Emila University, Modena, Italy; 2 Department of Medicine (Respiratory Division), University of British Columbia, Vancouver, British Columbia, Canada; 3 Department of Radiology, University of British Columbia, Vancouver, British Columbia, Canada; 4 UBC James Hogg Research Center, St. Paul’s Hospital, Vancouver, British Columbia, Canada; University of Cape Town Lung Institute, South Africa

## Abstract

**Background:**

With the widespread use of anti-retroviral therapy (ART), individuals infected with human immune deficiency virus (HIV) are increasingly experiencing morbidity and mortality from respiratory disorders. However, the prevalence or the risk factors associated with emphysema and bronchiolitis are largely unknown.

**Methods:**

Thoracic computed tomography (CT) scans were performed in 1,446 patients infected with HIV who were on ART and who attended a tertiary care metabolic clinic (average age 48 years and 29% females). Detailed history and physical examination including anthropometric measurements were performed. Complete pulmonary function tests were performed in a subset of these patients (n = 364). No subjects were acutely ill with a respiratory condition at the time of CT scanning.

**Findings:**

Nearly 50% of the subjects had CT evidence for emphysema, bronchiolitis or both with 13% (n = 195) showing bronchiolitis, 19% (n = 274) showing emphysema and 16% (n = 238) revealing both. These phenotypes were synergistically associated with reduced regular physical activity (p for interaction <.0001). The most significant risk factors for both phenotypes were cigarette smoking, intravenous drug use and peripheral leucocytosis. Together, the area-under-the curve statistics was 0.713 (p = 0.0037) for discriminating those with and without these phenotypes. There were no significant changes in lung volumes or flow rates related to these phenotypes, though the carbon monoxide diffusion capacity was reduced for the emphysema phenotype.

**Interpretation:**

Emphysema and bronchiolitis are extremely common in HIV-infected patients who are treated with ART and can be identified by use of thoracic CT scanning.

## Introduction

The widespread use of highly active anti-retroviral therapy (ART) has dramatically increased the longevity of patients infected with the human immune deficiency virus (HIV), with mortality rates falling from 30 at its peak to only 1–3 per 100 person-years. [1], [2] Many individuals with HIV infection in Western Countries are now living into their 60’s. [3] This improved survival has been driven largely by a marked reduction in deaths from acquired immune deficiency syndrome (AIDS)-defining conditions such as opportunistic infections and AIDS-related malignancies. [4] However, the impact of ART on non-AIDS defining illnesses has been less impressive. Currently, these conditions account for 50% to 60% of all deaths. [5] Interestingly, most of these non-AIDS defining conditions are senescence-related and in HIV appear to occur two to three decades earlier than those observed in the non-HIV infected populations. [6], [7] While the effects of HIV on two of the leading non-AIDS defining causes of mortality, cardiovascular disease and solid organ malignancies, have been carefully described and studied, [6]–[9] the relation of HIV with chronic obstructive pulmonary disease (COPD), another common senescence-related condition, remains poorly understood. Previous epidemiological studies suggest that the risk of COPD is accelerated in individuals with HIV. However, these studies have been relatively small in size and scope or failed to properly phenotype these patients. [10]–[12] The principal aim of the present study was to use thoracic computed tomography (CT) imaging to determine the prevalence of bronchiolitis and emphysema, which is one of the major morphologic phenotypes of COPD and to describe risk factors associated with these CT phenotypes in a large group of HIV infected individuals who were well treated with ART.

## Methods

### Cohort Description

This is a prospective observational study of all individuals infected with HIV who attended an outpatient HIV metabolic clinic at University of Modena and Reggio Emilia, Italy between January 2006 and September 2012 and who underwent CT scanning for cardiac risk assessment. No subject had a clinical diagnosis of COPD, bronchiectasis or any other chronic respiratory condition at the time of evaluation. The project received approval from the regional institutional ethics review committee at University of Modena (Comitato Etico Provinciale) and all subjects provided a written consent to participate in the study. Demographic data, clinical history, anthropometric assessment, blood tests, and pulmonary function tests (for those who were enrolled in 2012) were obtained in these subjects. Inclusion criteria were: serologically documented HIV-1 infection, age more than 18 years, and at least 18 months of ART exposure. Demographic and clinical data included at the time of CT scanning were: age, sex, current smoking status and intensity of smoking (i.e., the number of cigarettes consumed per day for current smokers), previous use of (illicit) intravenous drugs, a prior history of pneumonia (of any kind), and a history of *pneumocystis jiroveci* pneumonia (PJP). Moreover, through an in-person interview, we ascertained the subject’s cumulative exposure to major antiretroviral drug classes, including protease inhibitors (PI) and nucleoside reverse transcriptase inhibitors (NRTI), and their physical activity was determined on the basis of whether or not the respondents performed 2 or more hours of exercise per week, which is the minimal level of exercise recommended for achieving health benefits. [13] Anthropometric data included: body mass index (BMI), visceral abdominal adipose tissue volume (VAT) and subcutaneous adipose tissue (SAT), which were obtained using a single-slice abdominal CT scan at the level of the L4 vertebra as per standard protocol. [14] Pulmonary function testing was conducted using Jaeger Flow Screen spirometer and a Jaeger Master Screen plethysmography (RA.Med srl Medical technology) according to the ATS/ERS recommendations. [15].

### Thoracic CT Scans

All patients underwent CT imaging with a volume CT 64-slice scanner (GE Medical Systems, Milwaukee, Wisconsin, USA). All images were obtained during a single breath hold using 320 mAs and 140 kV. A section thickness of 2.5 mm, a field of view of 20 cm, and a matrix of 512×512 were used to reconstruct the raw image data, yielding a nominal pixel size of 0.39 mm^2^ and a voxel of 0.4 mm^3^. Until January 2012, images were reconstructed with a section thickness of 2.5 mm and a display field of view of 20 cm. Image acquisition commenced above the bronchial carina to the cardiac apex. From February 2012 and through completion of enrollment in September 2012, Z axis coverage was extended from the lung apices to the lung bases with reconstruction with a wide display field of view (FOV) of 40 cm.

Images were reviewed by 3 radiologists by consensus reading using an offline CT workstation (AW 4.4, GE Healthcare, Milwaukee, WI). Images were evaluated for findings of emphysema, and bronchiolitis as defined by the Fleischner Society glossary of terms. [13] We did not include “tree in bud” nodularity in the definition of bronchiolitis to reduce the likelihood of inclusion of infectious causes of bronchiolitis. Based on a modified method of Kazerooni et al, [16] semi-quantitative scores were assigned to all scans. A score (between 0 to 4) was assigned to each of 6 lobes (for a maximum score of 24) to describe the burden of lung emphysema manifested as bullae, centrilobular or paraseptal lesions. A total score of 0 indicated the absence of emphysema; a score of 1–2 indicated a mild degree of emphysema; 3–4 denoted a moderate amount of emphysema; and >4 indicated severe emphysema. Bronchial abnormalities were detected by noting bronchiolitis, manifesting as centrilobular nodules or tree in bud. Respiratory bronchiolitis, when present, was semi-quantitatively graded into 4 categories: 0 to 4 according to the amount of centrilobular micronodules and patchy ground-glass opacity, with or without fine fibrosis.

### Laboratory Studies

Blood was collected using standard venipuncture methods after an overnight fast. CD4 T-lymphocyte count, nadir CD4 T-cell count, quantitative plasma HIV-1 RNA (Real Time Abbott Molecular Inc., Des Plaines, IL, USA), C-reactive protein (CRP) was measured in the blood samples using standard assays with 1.1 mg/L as the lower limit of detection. These measurements were performed at the metabolic clinic, the University of Modena and Reggio Emilia, Italy. Pulmonary function tests were performed according to ATS/ERS standards with the subjects seated. [17] Inhaled salbutamol (400 ug) was provided when subjects demonstrated airflow limitation as defined by forced expiratory volume in one second (FEV1) to forced vital capacity (FVC) was less than 70%. Thus, FEV_1_ and FVC used in this study represent post-bronchodilator values for those who demonstrated airflow limitation and pre-bronchodilator values when they did not. Diffusing capacity of the lung for carbon monoxide (D_L_CO) was measured using the single breath method in accordance with the ATS/ERS recommendations. [18] D_L_CO values were not corrected for hemoglobin or carboxyhemoglobin. For normative values, we used Qunajer’s equation for FEV_1_ and FVC [19], Coats’ equation for D_L_CO [20], and Stocks’ equation for the other lung volumes [21]. Subjects who were experiencing acute respiratory illness were excluded from these tests.

### Statistical analysis

The cohort was first divided into 4 groups based on the CT findings: “no lung disease”, “emphysema”, “bronchiolitis” and “emphysema plus bronchiolitis”. The emphysema group contained CT scans that had an emphysema score 1 or greater, while the bronchiolitis group contained scans with a bronchiolitis score of 2 or greater. Continuous variables were compared across these groups using a one-way ANOVA with Bonferroni post hoc testing for pairwise comparisons. Dichotomous variables were compared across the groups using a chi-square test or a Fisher’s exact test (where appropriate) with appropriate degrees of freedom. For analytic purposes, emphysema was classified into no (score of 0), mild (1–2), moderate (3–4), and severe (>4) groups and bronchiolitis was divided into no or trivial (scores of 0 or 1), mild (2), moderate (3) and severe (4+) groups. A dose relationship was determined for each of the clinical variables across the emphysema or bronchiolitis severity gradient using the Mantel-Haenszel test for trend. To adjust for confounding, we employed multivariate regression modeling. To create the most parsimonious, yet statistically robust model, we used Akaike’s Information Criteria (AIC) for variable selection. The variables that yielded the smallest AIC values were included in the final model. To determine the possible clinical utility of knowing these risk factors in discriminating those with CT changes from those without, we constructed an area under the curve-receiver operating characteristics (AUC-ROC) analysis. The variable with the highest AUC-ROC in the univariate model was chosen as “model 1” (base model). To this base model, the variable with the second highest AUC-ROC was added, creating “model 2”. To model 2, the variable with the 3^rd^ highest AUC-ROC was added, creating “model 3”. This process continued until all variables were exhausted or until the incremental p-value was no longer significant for 2 successive models. P values less than 0.05 (corrected for multiple comparisons) were considered significant and all analyses were conducted with SAS version 9.3 (Carey, N.C.).

## Results

### Overall Clinical Characteristics

**Figure 1:**
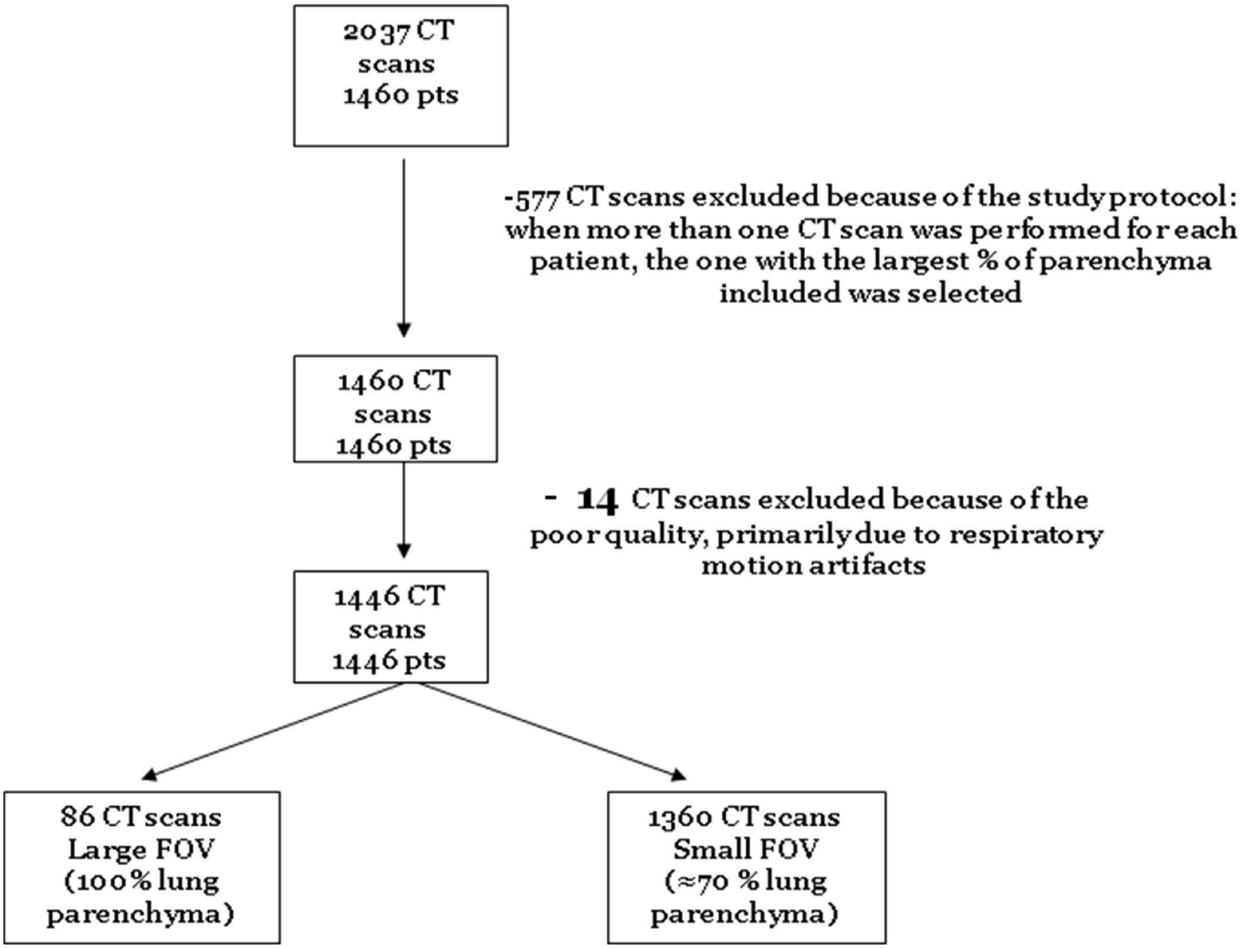
Flow Diagram of Patient Selection.

**Figure 2:**
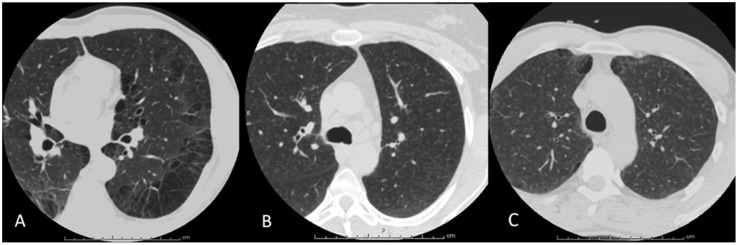
A Representative Image of a Subject with Emphysema (A), a Subject with Bronchiolits (B), and a Subject with both Emphysema and Bronchiolitis (C). A) A transverse axial image of a 48 year old HIV-infected male, displaying changes of severe emphysema as manifested by large bullae, as well as paraseptal and centrilobular regions of low attenuation. B) An axial image reconstructed with a small field of view in a 42 year old HIV-infected female, demonstrating changes of moderate respiratory bronchiolitis with multiple centrilobular nodules scattered throughout both lungs. C) A transverse axial image of a 44 year old HIV-infected male shows changes of both moderate emphysema and bronchiolitis.

There were 1,460 patients subjects who underwent 2,037 CT scans during the study period. We excluded duplicate scans, and 14 technically unsuitable scans (owing to motion artifact) for analysis, leaving 1,446 subjects (n = 417; 28.8% females) who had had at least one CT scan that was suitable for analysis (see Figure 1 for flow diagram). Of these, 258 subjects underwent full lung scans and 1,188 had cardiac CT scans. All patients were receiving ART at the time of assessment. The mean age of the subjects was 48.4±7.6 years and the mean body mass index (BMI) was 23.7±3.8 kg/m^2^. HIV was well controlled at the time of assessment with 94% (n = 1,356) of the subjects having an undetectable plasma viral load. Their mean CD4 count was 612±279 cells/mm^3^. The average duration of HIV infection, which was calculated by taking the difference between the date of study entry and date of HIV diagnosis, was 202±75 months. Approximately 40% (n = 558) of the subjects were current smokers and 28% (n = 403) had a significant history of previous intravenous drug use; none were active users at the time of CT assessment. CT scans demonstrated evidence for bronchiolitis, emphysema or both in 49% of the subjects (n = 707): 13% (n = 195) showed bronchiolitis, 19% (n = 274) demonstrated emphysema and 16% (n = 238) revealed both. The prevalence of emphysema (as defined by presence of qualitative emphysema score of 1 or greater) was 41% (n = 105) in those who had a full lung scan versus 34% (n = 406) in those who had a cardiac scan (p = 0.0470). Clinical characteristics stratified according to CT changes are summarized in Table 1. Representative images of a subject with emphysema, a subject with bronchiolitis and a subject without these CT changes are shown in Figure 2.

**Table 1:** Clinical Characteristics of the 1,446 Consecutive Subjects With HIV Infection According to COPD Changes on CT Scans.

Group	No lung disease	Bronchiolitis	Emphysema	Emphysema + Bronchiolitis	Global p value
**No of Patients**	739 (51%)	195 (13%)	274 (19%)	238 (16%)	–
**Women**	251 (34%)	72 (37%)	46 (17%)	48 (20%)	<.0001
**Age (years)**	47.4±7.8	47.1±6.2	51.2±7.8*	49.4±7.1* †	<.0001
**Current Smokers**	176 (24%)	110 (58%)*	95 (35%)* ‡	177 (77%)* † ‡	<.0001
**>10 cigs/day**	76 (10%)	61 (31%)‡	57 (21%)‡	131 (55%)‡	<.0001
**Intravenous Drug Use**	137 (19%)	63 (32%)*	98 (36%)*	105 (44%)*	<.0001
**HIV exposure (months)**	192±76	212±76*	211±68*	217±74*	<.0001
**Suppressed viral load**	691 (94%)	179 (92%)	260 (95%)	226 (95%)	0.4650
**Nadir CD4 (cells/mm^3^)**	206±152	203±176	175±151*	187±181	0.0309
**Current CD4 (cells/mm^3^)**	603±258	633±286	585±285	658±323†	0.0180
**Current Use of PI**	433 (59%)	151 (55%)	121 (62%)	143 (61%)	0.4962
**Current Use of NNRTI**	271 (37%)	102 (37%)	62 (32%)	80 (34%)	0.5123
**Current Use of NRTI**	621 (84%)	244 (89%)	165 (85%)	196 (82%)	0.1384
**Body mass index (kg/m^2^)**	24.1±3.9	23.0±3.4*	23.8±3.7	22.8±3.3* †	<.0001
**Subcutaneous Adipose Tissue (cm^2^)**	167±99	140±84*	142±89*	125±79*	<.0001
**Visceral Adipose Tissue (cm^2^)**	132±75	115±58*	147±92* ‡	125±70†	<.0001
**Prior PJP infection**	46 (7%)	9 (5%)	28 (12%)	21 (10%)	0.0743
**Prior TB infection**	11 (1%)	4 (2%)	8 (3%)	7 (3%)	0.3648
**Prior Pneumonia**	54 (8%)	25 (15%)*	41 (17%)*	39 (18%)*	0.0001
**History of Asthma**	5 (1%)	6 (4%)	1 (0%)	3 (1%)	0.0241
**CRP (>1.1 mg/L)**	322 (46%)	87 (48%)	120 (47%)	130 (59%)* ‡	0.0138
**White blood cell (cells/µL)**	5872±1687	6095±1902	6302±1931*	7498±2420* † ‡	<.0001
**Regular Physical activity** ††	350 (49%)	72 (38%)*	115 (43%)	89 (39%)*	0.0088

Dichotomous data are presented as number of individuals (% column totals) and continuous variables are presented as mean±SD.

*p<.05 vs no lung disease, following Bonferroni correction.

‡ p<.05 vs bronchiolitis, following Bonferroni correction.

† p<.05 vs emphysema, following Bonferroni correction.

†† 2 or more hours of physical activity per week.

Abbreviations: cigs, cigarettes; CRP, C-reactive protein; HIV, human immune deficiency; NNRTI, non-nucleoside reverse transcriptase inhibitors; NRTI, nucleoside reverse transcriptase inhibitors; PJP, pneumocystis jiroveci pneumonia; PI, protease inhibitors; TB, tuberculosis.

There were commonly shared risk factors for both bronchiolitis and emphysema. These included cigarette smoking, intravenous drug use, duration of HIV infection, reduced BMI or SAT, a prior history of pneumonia, and elevated peripheral leukocyte count or CRP levels (see Tables 2 and 3). There were some notable discordances in risk factors between the two CT phenotypes. For instance, increasing age and male sex were significantly related to emphysema but not to bronchiolitis. Similarly, prior history of PJP infection was a risk factor for emphysema but not for bronchiolitis. Conversely, reduced visceral adiposity was a risk factor for bronchiolitis but not for emphysema. Importantly, both emphysema and bronchiolitis severity scores were associated with reduced regular physical activity, indicating functional impairment (see Tables 2 and 3).

**Table 2:** Clinical Risk Factors and Health Outcomes for Emphysema.

Emphysema Score (severity)	0 (none)	1–2 (mild)	3–4 (moderate)	>4 (severe)	P value for trend
**No of Patients**	935 (65%)	189 (13%)	130 (9%)	192 (13%)	–
**Women**	323 (35%)	34 (18%)	35 (27%)	25 (13%)	<.0001
**Age (years)**	47.4±7.5	50.1±8.1	49.5±7.4	51.0±7.1	<.0001
**Current Smokers**	286 (31%)	77 (42%)	76 (60)	119 (63%)	<.0001
**Intravenous Drug Use**	200 (21%)	65 (34%)	49 (38%)	89 (46%)	<.0001
**HIV exposure (months)**	196±75	200±78	210±71	231±73	<.0001
**Nadir CD4 Count (cells/mm^3^)**	206±157	172±144	186±187	186±169	0.0190
**Current CD4 Count (cells/mm^3^)**	609±264	597±299	605±320	652±302	0.1518
**Body mass index (kg/m^2^)**	23.9±3.8	24.0±3.5	23.1±3.9	22.8±3.3	0.0002
**Subcutaneous Adipose Tissue (cm^2^)**	162±97	145±87	133±84	124±83	<.0001
**Visceral Adipose Tissue (cm^2^)**	129±72	137±86	137±86	135±79	0.1315
**Prior PJP infection**	55 (7%)	16 (9%)	16 (14%)	17 (10%)	0.0289
**Prior Pneumonia**	79 (10%)	27 (16%)	20 (17%)	33 (19%)	<0.001
**CRP (>1.1 mg/L)**	409 (46%)	87 (49%)	66 (54%)	97 (55%)	0.0120
**WBC (/µL)**	5915±1732	6394±1819	6834±2320	7295±2495	<0.001
**FEV1 (% predicted)**	105±16	109±15	100±19	104±22	0.5937
**FEV1/FVC (%)**	79.8±6.5	80.8±5.9	77.5±6.9	76.2±7.3	0.0011
**D_LCO_ (% predicted)**	77.3±15.9	76.2±18.1	72.8±14.2	65.2±12.3	<.0001
**Bronchiolitis Score**	0.80±0.81	1.22±0.96	1.6±0.95	1.4±0.96	<.0001
**Regular Physical activity** *	423 (47%)	82 (45%)	56 (44%)	65 (35%)	0.0060

Dichotomous data are presented as number of individuals (% column totals) and continuous variables are presented as mean±SD.

*2 or more hours of physical activity per week.

Abbreviations: CRP, C-reactive protein; D_LCO_, diffusing capacity of lung for carbon monoxide or transfer factor; FEV1, forced expiratory volume in one second; FVC, forced vital capacity; HIV, human immune deficiency; PJP, pneumocystis jiroveci pneumonia.

**Table 3:** Clinical Risk Factors and Health Outcomes for Bronchiolitis.

Bronchiolitis Score (severity)	0–1 (none or trivial)	2 (mild)	3 (moderate)	4+ (severe)	P value for trend
**No of Patients**	1,013 (70%)	362 (25%)	58 (4%)	13 (1%)	–
**Women**	297 (29%)	104 (29%)	12 (21%)	4 (31%)	0.3817
**Age (years)**	48.4±8.0	48.5±7.0	47.0±5.8	48.4±6.6	0.5201
**Current Smokers**	271 (27%)	225 (64%)	50 (89)	12 (92%)	<.0001
**Intravenous Drug Use**	235 (23%)	130 (36%)	30 (52%)	8 (62%)	<.0001
**HIV exposure (months)**	197±76	210±71	236±71	230±63	<.0001
**Nadir CD4 Count (cells/mm^3^)**	198±152	200±182	164±167	179±111	0.3589
**Current CD4 Count (cells/mm^3^)**	598±265	654±310	622±298	548±243	0.0438
**Body mass index (kg/m^2^)**	24.1±3.9	23.1±3.3	22.1±3.2	22.0±3.5	<.0001
**Subcutaneous Adipose Tissue (cm^2^)**	161±97	135±82	118±83	98±72	<.0001
**Visceral Adipose Tissue (cm^2^)**	136±80	122±65	113±67	106±52	0.0003
**Prior PJP infection**	74 (8%)	20 (6%)	8 (15%)	2 (15%)	0.0823
**Prior Pneumonia**	95 (11%)	57 (18%)	7 (13%)	0 (0%)	0.0074
**CRP (>1.1 mg/L)**	442 (47%)	178 (53%)	30 (56%)	9 (75%)	0.0443
**WBC (/µL)**	5992±1768	6723±2293	7660±2269	7509±2433	<.0001
**FEV1 (% predicted)**	104.7±16.3	106.2±18.3	104.7±20.1	96.2±16.1	0.8522
**FEV1/FVC (%)**	79.1±6.9	79.4±6.3	78.6±7.4	75.8±7.4	0.8539
**D_LCO_ (% predicted)**	76.0±16.5	73.3±16.0	67.9±11.4	71.2±13.3	0.0475
**Emphysema Score**	0.52±0.97	1.05±1.20	1.55±1.17	1.85±0.97	<.0001
**Regular physical activity** *	465 (47%)	149 (43%)	11 (20%)	1 (8%)	<.0001

Dichotomous data are presented as number of individuals (% column totals) and continuous variables are presented as mean±SD for normally distributed variables or median (interquartile range) for non-normally distributed variables.

*2 or more hours of physical activity per week.

Abbreviations: CRP, C-reactive protein; D_LCO_, diffusing capacity of lung for carbon monoxide or transfer factor; FEV1, forced expiratory volume in one second; FVC, forced vital capacity; HIV, human immune deficiency; PJP, pneumocystis jiroveci pneumonia.

### Relative Importance of Risk Factors

With both CT phenotypes, cigarette smoking was the single most important risk factor (see Tables S1 and S2). However, cigarette smoking was associated with a higher r^2^ value for bronchiolitis compared with emphysema (0.152 versus 0.063), suggesting a more prominent role of cigarette smoking in the bronchiolitis phenotype. Peripheral leukocytosis was also strongly related to both of these phenotypes and accounted for approximately 5% and 6% of the variances in the bronchiolitis and emphysema scores, respectively, and intravenous drug use accounted for 3% of the variance in the bronchiolitis and 4% of the variance in the emphysema scores (see tables S1 and S2). Increasing age and sex (each) accounted for 3% of the emphysema score variance but neither contributed significantly to the bronchiolitis score. The other risk factors contributed very little to the overall variance in the CT phenotype scores.

### CT Phenotypes and Physiology and Health Outcomes

**Figure 3:**
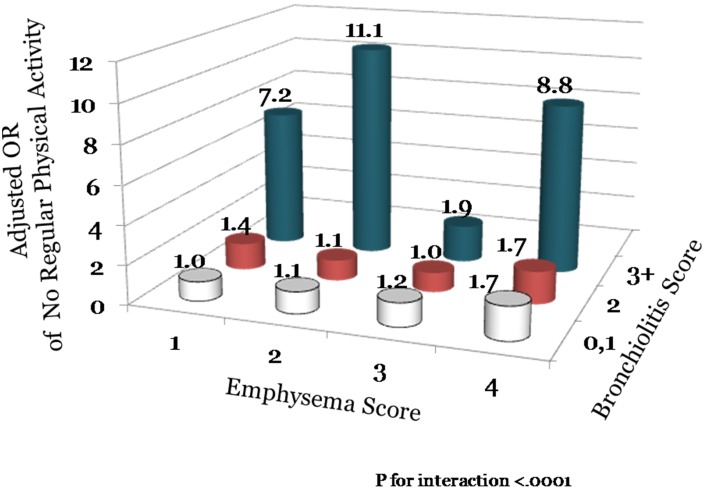
The Synergistic Interaction of Emphysema with Bronchiolitis Scores on The Risk of Reduced Regular Physical Activity.

Lung function measurements were performed in 364 subjects. Of these, 9.6% demonstrated spirometric evidence for COPD with 51% having GOLD grade 1, 40% having GOLD grade 2 and 9% GOLD grade 3 disease. Interestingly, 37% of the subjects had reduced diffusing capacity of the lung for carbon monoxide (D_LCO_) defined as D_LCO_ less than 80% of predicted value. The emphysema phenotype independent of bronchiolits (see Table 2) or emphysema plus bronchiolitis phenotype (see Table 4) was associated with impaired gas exchanged. With increasing severity of emphysema, there was also a progressive reduction in the FEV1/FVC ratio (see Table 2). However, even in the group with very severe emphysema (score of >4), the vast majority of subjects had an FEV1/FVC ratio within normal limits (>0.7). Neither the bronchiolitis nor emphysema phenotype was associated with impaired flow rates or representative lung volumes (Table 4). However, despite the absence of significant physiological perturbations, both emphysema and bronchiolitis was associated with impairments in physical activity (see Tables 2 and 3). There was a synergistic interaction of emphysema with bronchiolitis scores on the risk of impaired physical activity (p for interaction, <.0001; see Figure 3). Compared with those without any emphysema or bronchiolitis, those with severe emphysema (>4) and moderate to severe bronchiolitis (3+) had a nearly 9 fold increase in the odds of impaired physical activity (adjusted odds ratio, OR, 8.82; 95% CI, 2.02 to 38.6; p = 0.0408).

**Table 4:** Pulmonary Function Test Results Of 364 HIV Infected Subjects Stratified According To CT Based Emphysema Severity.

Emphysema Score (severity)	0 (none)	1–2 (mild)	3–4 (moderate)	>4 (severe)	P value for trend
**No of Patients**	207	58	34	65	–
**FEV1 (% predicted)**	106±17	106.8±17	104±11	101±21	0.0438
**FVC (% predicted)**	109.8±37.5	107.1±14.5	108.0±9.7	109.5±17.8	0.8561
**FEV1/FVC (%)**	80.2±6.6	80.4±6.0	78.7±4.3	73.8±7.7	<.0001
**RV (% predicted)**	137.0±21.3	142.6±18.0	147.9±29.1*	146.2±25.2	0.0007
**TLC (% predicted)**	114.2±13.5	112.7±11.8	113.7±11.7	121.1±13.2	0.0180
**RV/TLC (%)**	40.6±8.0	39.0±6.2	39.9±4.3	42.4±8.2	0.3505
**D_LCO_ (% predicted)**	78.5±13.0	78.7±15.6	72.7±15.3	64.2±12.9	<.0001
**D_LCO_/VA (% predicted)**	90.9±14.7	90.7±16.6	85.3±15.9	72.7±13.8* ‡	<.0001

Dichotomous data are presented as number of individuals (% column totals) and continuous variables are presented as mean±SD for normally distributed variables.

*p<.05 vs no lung disease, following Bonferroni correction.

‡ p<.05 vs bronchiolitis, following Bonferroni correction.

Abbreviations: FEV1, forced expiratory volume in one second; FVC, forced vital capacity, TLC, total lung capacity; RV, residual volume; D_LCO_, diffusing capacity of lung for carbon monoxide or transfer factor; D_LCO_/VA, D_LCO_ corrected for alveolar volume or transfer coefficient.

### Predictors of CT Phenotypes

To determine which risk factors could predict the occurrence of bronchiolitis or emphysema on thoracic CT scans, we calculated AUC-ROC values for a select number of clinical and demographic variables (see Table 5). Smoking alone had an AUC-ROC value of 0.656 (95% CI, 0.631 to 0.680). Adding peripheral leukocyte count to the smoking variable increased the AUC-ROC to 0.689 (p = 0.0004). Inclusion of a history of intravenous drug use to smoking and peripheral leukocyte count increased the AUC-ROC to 0.713 (p = 0.0037). Addition of age to this model further increased the AUC-ROC value to 0.730 (p = 0.0360). Although both BMI and sex were significantly related to CT phenotypes, their addition did not significantly improve the discriminatory properties of the model (see Table 5).

**Table 5:** Risk Predictors for Bronchiolitis or Emphysema Detected on CT Scans.

Model	Variables	Crude OR (95% CI)	ROC (95% CI)	Multivariate OR (95% CI)*	Incremental ROC (95%)†	p‡
**1**	**Current Smoker**	3.86 (3.08,4.85)	0.656 (0.631, 0.680)	3.48 (2.58, 4.71)	0.656 (0.631, 0.680)	–
**2**	**WBC (per 10^3^ cells/mm^3^ increase)**	1.23 (1.15, 1.32)	0.614 (0.579, 0.648)	1.13 (1.05, 1.22)	0.689 (0.656, 0.721)	0.0004
**3**	**Intravenous Drug Use**	2.65 (2.09, 3.37)	0.595 (0.573, 0.618)	2.14 (1.56, 2.94)	0.713 (0.681, 0.744)	0.0037
**4**	**Age (per 10 year increase)**	1.42 (1.24, 1.64)	0.577 (0.548, 0.606)	1.63 (1.35, 1.99)	0.730 (0.699, 0.760)	0.0360
**5**	**BMI (per 5 kg/m^2^ decrease)**	1.38 (1.20, 1.60)	0.568 (0.538, 0.598)	1.43 (1.18, 1.74)	0.733 (0.703, 0.764)	0.3145
**6**	**Men**	1.68 (1.33, 2.11)	0.552 (0.529, 0.576)	2.03 (1.46, 2.81)	0.743 (0.713, 0.773)	0.0679

Abbreviations: BMI, body mass index; CI, confidence interval; OR, odds ratio; ROC, receiver operating characteristics; WBC, white blood cell;

*adjusted for all the variables listed in this table in a multivariate logistic regression model (see Methods for detail).

† Incremental ROC represents area under the curve obtained by adding the previous row variable to the current row variable in a multivariate logistic regression model (see Methods for detail). For example, model 2 incremental ROC and p values are obtained by comparing a logic regression model that contains WBC and current smoker variables to that which contains only current smoker variable (i.e. model 1).

‡ p value is obtained by comparing the incremental ROC value of the current row to the previous row ROC value.

## Discussion

To our knowledge, this is the largest epidemiological study using CT scanning to describe the prevalence of emphysema and bronchiolitis and their risk factors in HIV infected individuals treated with ARTs. Although the individuals were not selected on the basis of pulmonary risk factors or symptoms, strikingly, nearly 50% of these patients whose mean age was only 48 years had imaging evidence for bronchiolitis, emphysema or both and those who had these CT phenotypes reported reduced physical activity, suggesting exercise impairment. The single most important risk factor for the presence of bronchiolitis or emphysema was cigarette smoking. Additional (independent) risk factors included peripheral leukocytosis, and a history of intravenous drug use. Together, these data suggest a complex interplay of environmental irritant exposure with persistent systemic inflammation that may enhance and accelerate lung injury, leading to bronchiolitis and emphysema HIV infected individuals who are on ART.

Why these CT phenotypes develop in relatively young HIV individuals is not known with any certainty. One suggested pathway is the additive effect of lung injury (from prior infection, smoking, and illicit drug exposure) and the viral infection which modifies the immune system responses, leading to chronic inflammatory and senescent changes in major organs, such as the lungs, liver and bone marrow. It is now well known that despite long-term suppressive therapy with ART, chronic systemic inflammation persists, which may amplify and accelerate the pathogenesis of COPD in these individuals. Consistent with this theory, we found that individuals with leukocytosis and elevated CRP were more likely to demonstrate emphysema and bronchiolitis. Alternatively, some have shown that HIV infected individuals experience premature senescence and multiple organ pathologies. [22] They also demonstrate accelerated attrition of telomeres in replicative cells such as certain leukocytes and progenitor cells. [23], [24] In experimental models, reduction in telomere length has been shown to increase the risk of emphysema in the presence of chronic cigarette smoke exposure. [25] It is also notable that direct HIV variables such as current CD4 count and plasma viral load had very little impact in predicting the occurrence of emphysema or bronchiolitis most likely because these patients were treated with ART and had a good clinical and virologic response. These findings suggest that HIV related COPD in the ART-era is driven predominantly by premature aging and lifestyle factors (e.g. smoking, intravenous drug use).

Although there were commonalities in risk factor for emphysema and bronchiolits, there were risk factors unique for these two phenotypes. For example, increasing age was a risk factor for emphysema but not bronchiolitis. These data are consistent with the emerging concept that in most smokers, COPD changes first start in the small airways with cigarette smoking and then progress over time into the gas exchanging units, resulting in emphysema. [26] In this regard, CT scanning that we did might not have been sufficiently sensitive to detect early subtle changes in the small airways. Another notable difference was the relationship of BMI to the CT phenotypes. While there was a clear inverse relationship between BMI and the risk of emphysema, its relation with bronchiolitis was much weaker. The reasons for this are obscure and will require additional investigations.

While CT scanning is extremely helpful for non-invasive morphologic phenotyping and detection of early disease, it is expensive and fraught with certain adverse effects and as such should be used with caution. Our study indicates that knowledge of certain risk factors may increase the chance of finding significant abnormalities on CT scanning. Smoking status by itself has relatively poor discrimination. The addition of peripheral leukocyte count, a history of intravenous drug use and age to smoking status, however, significantly increases discrimination from “poor” to the “fair” to “good” range. Further work will be needed to optimize classifiers to accurately and cost-effectively identify HIV infected patients who will benefit most from “screening” CT scans.

There were limitations to this study. First, we did not obtain pulmonary function measurements in the entire cohort. As such, the study may have lacked sufficient power to determine the relationship of CT phenotypes to airflow limitation in HIV infected patients. Second, the use of coronary calcium quantification CT examinations in some subjects precluded full visualization of lung apices, diaphragm, and lung peripheries. However, a previous study has shown an excellent (r = 0.93) correlation in the extent of emphysema between cardiac and full lung scans, proving some assurances. [27] It is also possible that the exclusion of peripheral and apical areas of the lung may have led to the underestimation of the radiographic burden of COPD in these patients. Thus, our estimates of bronchiolitis and emphysema in HIV infected individuals are likely conservative. Third, our study was not designed to determine the long-term effects of COPD phenotypes in HIV infected patients. Longer studies will be needed to answer this critically important question. Fourth, as this was a non-interventional observational study, we did not evaluate the effects of pulmonary therapies in these patients. Finally, it should be noted that the COPD phenotypic changes observed in the present study are all based on CT imaging and not on histology. Thus, it is possible that the pathogenesis and natural history of “emphysema” and “bronchiolitis” in HIV may be dissimilar to those related to smoking in the non-HIV patients. Fourth, although none of the subjects in this study were suffering an acute respiratory tract infection at the time of CT assessment, we cannot be certain that bronchiolitis observed on CT scans represents small airways disease of COPD. As our study did not contain any expiratory scans, we could not assess gas trapping. Future studies will be required to fully evaluate the causes of bronchiolitis in HIV infected subjects.

Notwithstanding these and other limitations, the findings from the present study have important clinical and public health implications. First, the prevalence of bronchiolitis and emphysema is huge in the HIV infected individuals (∼50%) and manifests much earlier than in the general population (40’s versus 60’s). Spirometry, however, is insensitive in detecting significant abnormalities in this patient population; CT scans are needed. Second, the three leading risk factors for these CT phenotypes are: smoking, peripheral leukocytosis and intravenous drug use in HIV infected patients. In view of the high rates of smoking and intravenous drug use among these patients, these data emphasize the critical importance and pre-eminence of addiction treatment in confronting the lung disease epidemic in these patients. These data also highlight the likely importance of chronic systemic inflammation in the pathogenesis of smoking-related lung disease in HIV infected patients. Additional work will be needed to confirm this hypothesis. Third, the use of simple clinical and demographic data consisting of 4 risk factors (age, smoking status, intravenous drug use and peripheral leukocyte count) may enable more rational and cost-effective use of CT scanning for COPD detection in HIV infected patients. CT scanning may also assist clinicians in identifying source of unexplained dyspnea and physical activity impairment in these patients. Additional work will be needed to validate this notion.

In summary, this large study has demonstrated that morphologically diagnosed emphysema in ∼35%, of HIV infected patients who do not have a primary respiratory complaint. The COPD epidemic will grow in strength and number as HIV infection becomes a chronic illness. There is an urgent need to tackle the enormous burden of lung disease in these patients through better and more comprehensive management of their cigarette and illicit drug addiction, improved education, and additional research on mechanisms responsible for accelerated COPD in this group of patients.

## Supporting Information

Table S1 **Clinical Variables Significantly Related to Emphysema Severity.** The variables in this table were chosen based on the lowest AIC (Akaike’s Information Criteria) value, estimates the difference between a given model and the “true” model. The model with the smallest AIC among all competing models is deemed the best model (see Methods for detail). *β-coefficients were derived from a multivariate linear regression model that contained all of the variables listed in the table. †standardized coefficient estimates the change in the emphysema score (grouped as 0, 1–2, 3–4, >4) per 1 standard deviation increase for the continuous variables in a multivariate regression model. The above variables combined have an AIC value of −5.62 and adjusted R^2^ value of 0.19.
(DOC)

Table S2 **Clinical Variables Significantly Related to Bronchiolitis Severity.** The above variables were chosen based on the lowest AIC (Akaike’s Information Criteria) value, estimates the difference between a given model and the “true” model. The model with the smallest AIC among all competing models is deemed the best model (see Methods for detail). *β-coefficients were derived from a multivariate linear regression model that contained all of the variables listed in the table. †standardized coefficient estimates the change in the bronchiolitis score (grouped as 0, 1, 2, 3 or more) per 1 standard deviation increase for the continuous variables in a multivariate regression model. The above variables combined have an AIC value of −358.9 and adjusted R^2^ value of 0.21.
(DOC)
