# Supplementary material for: The Burden of Image Based Emphysema and Bronchiolitis in HIV-Infected Individuals on Antiretroviral Therapy
Source: PLoS One. 2014 Oct 29;9(10):e109027. doi: 10.1371/journal.pone.0109027 (PMC4212912; doi:10.1371/journal.pone.0109027)
Supplement: Table S1 — Clinical Variables Significantly Related to Emphysema Severity. The variables in this table were chosen based on the lowest AIC (Akaike’s Information Criteria) value, estimates the difference between a given model and the “true” model. The model with the smallest AIC among all competing models is deemed the best model (see Methods for detail). *β-coefficients were derived from a multivariate linear regression model that contained all of the variables listed in the table. †standardized coefficient estimates the change in the emphysema score (grouped as 0, 1–2, 3–4, >4) per 1 standard deviation increase for the continuous variables in a multivariate regression model. The above variables combined have an AIC value of −5.62 and adjusted R2 value of 0.19. (DOC) [file pone.0109027.s001.doc]

***Table S1:* Clinical Variables Significantly Related to Emphysema Severity**

| **Variables** | **Adjusted β-coefficient±SE^*^** | **Standardized Coefficient^†^** | **Adjusted R2** | **P-value** |
| --- | --- | --- | --- | --- |
| **Current Smoker** | 0.400±0.070 | 0.178 | 0.063 | <.0001 |
| **WBC (per 10^4^ cells/ µL increase)** | 1.059±0.019 | 0.196 | 0.063 | <.0001 |
| **Intravenous Drug Use** | 0.314±0.074 | 0.130 | 0.041 | <.0001 |
| **Age (per 10 yr increase)** | 0.231±0.047 | 0.156 | 0.030 | <.0001 |
| **Women** | -0.345±0.075 | -0.142 | 0.026 | <.0001 |
| **BMI (per 10 kg/m^2^ increase)** | -0.444±0.102 | -0.153 | 0.009 | <.0001 |
| **Current CD4 count (per 100 cell/mm3 increase)** | -0.030±0.013 | -0.075 | 0.001 | 0.0235 |
| **Visceral Adipose Tissue (per 100 cm^2^ increase)** | 0.076±0.051 | 0.056 | 0.001 | 0.1391 |

The above variables were chosen based on the lowest AIC (Akaike’s Information Criteria) value, estimates the difference between a given model and the “true” model. The model with the smallest AIC among all competing models is deemed the best model (see Methods for detail).

*β-coefficients were derived from a multivariate linear regression model that contained all of the variables listed in the table.

†standardized coefficient estimates the change in the emphysema score (grouped as 0,1-2, 3-4, >4) per 1 standard deviation increase for the continuous variables in a multivariate regression model.

The above variables combined have an AIC value of -5.62 and adjusted R^2^ value of 0.19
