# Supplementary material for: The Burden of Image Based Emphysema and Bronchiolitis in HIV-Infected Individuals on Antiretroviral Therapy
Source: PLoS One. 2014 Oct 29;9(10):e109027. doi: 10.1371/journal.pone.0109027 (PMC4212912; doi:10.1371/journal.pone.0109027)
Supplement: Table S2 — Clinical Variables Significantly Related to Bronchiolitis Severity. The above variables were chosen based on the lowest AIC (Akaike’s Information Criteria) value, estimates the difference between a given model and the “true” model. The model with the smallest AIC among all competing models is deemed the best model (see Methods for detail). *β-coefficients were derived from a multivariate linear regression model that contained all of the variables listed in the table. †standardized coefficient estimates the change in the bronchiolitis score (grouped as 0, 1, 2, 3 or more) per 1 standard deviation increase for the continuous variables in a multivariate regression model. The above variables combined have an AIC value of −358.9 and adjusted R2 value of 0.21. (DOC) [file pone.0109027.s002.doc]

***Table S2:* Clinical Variables Significantly Related to Bronchiolitis Severity**

| **Variables** | **Adjusted β-coefficient±SE^*^** | **Standardized Coefficient^†^** | **Adjusted R2** | **P-value** |
| --- | --- | --- | --- | --- |
| **Current Smoker** | 0.634±0.058 | 0.344 | 0.155 | <.0001 |
| **WBC (per 10^4^ cells/ µL increase)** | 0.536±0.150 | 0.120 | 0.043 | 0.0004 |
| **Intravenous Drug Use** | 0.245±0.061 | 0.123 | 0.036 | 0.0020 |
| **CRP (>1.1 mg/L versus ≤1.1 mg/L)** | 0.166±0.054 | 0.093 | 0.006 | 0.0021 |
| **BMI (per 10 kg/m^2^ increase)** | -0.172±0.093 | -0.076 | 0.023 | 0.0637 |
| **Current CD4 Count (per 100 cell/mm3 increase)** | -0.172±0.107 | -0.052 | 0.002 | 0.1071 |
| **Subcutaneous Adipose Tissue (per 100 cm^2^ increase)** | -0.045±0.036 | -0.046 | 0.026 | 0.2173 |

The above variables were chosen based on the lowest AIC (Akaike’s Information Criteria) value, estimates the difference between a given model and the “true” model. The model with the smallest AIC among all competing models is deemed the best model (see Methods for detail).

*β-coefficients were derived from a multivariate linear regression model that contained all of the variables listed in the table.

†standardized coefficient estimates the change in the bronchiolitis score (grouped as 0,1,2,3 or more) per 1 standard deviation increase for the continuous variables in a multivariate regression model.

The above variables combined have an AIC value of -358.9 and adjusted R^2^ value of 0.21
